# Vertebral artery dissection in a patient with migraine treated with calcitonin gene-related peptide monoclonal antibody: a case report and FAERS database analysis

**DOI:** 10.1186/s12883-024-04009-z

**Published:** 2025-01-02

**Authors:** Daiki Tokuyasu, Shungo Imai, Shih-Pin Chen, Keiko Ihara, Narumi Watanabe, Yoshikane Izawa, Jin Nakahara, Satoko Hori, Tsubasa Takizawa

**Affiliations:** 1 Department of Neurology, Keio University School of Medicine, 35 Shinanomachi, Shinjuku-ku, Tokyo, 160-8582 Japan; 2 Division of Drug Informatics, Keio University Faculty of Pharmacy, Tokyo, Japan; 3 Division of Translational Research, Department of Medical Research, Taipei Veterans General Hospital, Taipei, Taiwan; 4 Institute of Clinical Medicine, College of Medicine, National Yang Ming Chiao Tung University, Taipei, Taiwan; 5 Brain Research Center, National Yang Ming Chiao Tung University, Taipei, Taiwan; 6 Japanese Red Cross Ashikaga Hospital, Ashikaga, Japan

**Keywords:** Migraine, Cervical artery dissection, Vertebral artery dissection, CGRP, Galcanezumab, Case report

## Abstract

**Background:**

Migraine is associated with cervical artery dissection (CeAD). Calcitonin gene-related peptide (CGRP) is a multifunctional neuropeptide with vasodilatory effects. The use of anti-CGRP monoclonal antibodies (CGRP mAb) may affect cerebrovascular disease risk; however, no reports have associated CGRP mAb with CeAD.

**Case presentation and FAERS database analysis:**

We report a case of vertebral artery dissection in a 39-year-old woman with migraine treated with galcanezumab. We searched the number of cases where cerebral and cervical artery dissection were reported as adverse effects of CGRP mAb using the FDA Adverse Event Reporting System (FAERS) database. Six and ten such cases were reported regarding galcanezumab and CGRP mAbs use, respectively. The reporting odds ratios for galcanezumab and CGRP mAbs were elevated.

**Conclusion:**

Although migraine is reported to be associated with CeAD, the use of CGRP mAb might be related to CeAD and warrant further investigation.

**Supplementary Information:**

The online version contains supplementary material available at 10.1186/s12883-024-04009-z.

## Background

Migraine is one of the most common neurological disorders worldwide. Global migraine prevalence has recently been reported to be 14–15% [1]. Migraine, particularly migraine without aura, is associated with cervical artery dissection (CeAD) [2]. Some genetic correlations exist between migraine and CeAD, and it is assumed that vascular fragility may underlie both conditions [3]. Calcitonin gene-related peptide (CGRP) is a multifunctional neuropeptide targeted for the treatment of migraine and is known to have various effects, including a vasodilating effect [4]. Given the effects and expressions of CGRP, the side effects and off-target effects of anti-CGRP monoclonal antibodies (CGRP mAb) are recently of interest [5]. One of these concerns is that CGRP mAb may increase the risk of cerebrovascular disease. Similarly, CGRP mAb may affect CeAD, one of the cerebrovascular disorders; however, relevant reports are lacking.

We present a case of vertebral artery dissection that developed during CGRP mAb treatment for migraine without aura. To the best of our knowledge, there have been no reports of CGRP mAb and CeAD. We used the Food and Drug Administration (FDA) Adverse Event Reporting System (FAERS) database, a publicly available database containing spontaneous adverse event reports submitted to the FDA, to investigate the number of cerebral and cervical artery dissection events reported as adverse effects (AEs) of CGRP mAb.

## Case presentation

**Fig. 1:**
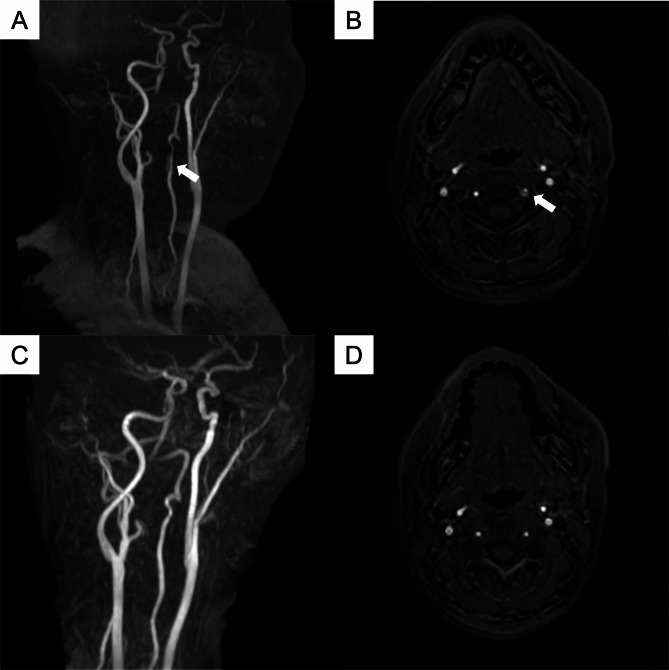
MRA findings. (**A**) Neck MRA revealed the stenosis of the V2 segment of the left vertebral artery (white arrow); (**B**) Axial MRA revealed “double lumen” of the left vertebral artery (white arrow); (**C**, **D**) Neck and axial MRA performed two months later revealed the improvement of the left vertebral artery dissection. MRA, magnetic resonance angiography

A 39-year-old woman was treated with galcanezumab since June 2021 for migraine without aura. She was a non-smoker and had no vascular risk factors such as hypertension, hyperlipidemia, or diabetes. Her family history did not include any cardiovascular or cerebrovascular diseases. After the 16th dose of galcanezumab, the patient developed neck pain on the left side followed by severe headaches that differed from her usual migraine headaches. This unusual headache was unilateral, non-pulsatile, and worsened with physical movement. Headache severity was 9–10/10 on a numerical rating scale. The patient had no traumatic or triggering events prior to the onset of headache. The patient did not report nausea, photophobia, or phonophobia. The patient visited her physician two weeks after experiencing persistent neck pain and headaches. Brain magnetic resonance imaging (MRI) revealed left vertebral artery stenosis, and the patient was referred to our department for further evaluation and treatment. Head and neck magnetic resonance angiography (MRA) showed a 15 mm-long vertebral artery dissection distal to the left V2 segment (Fig. 1). She had no physical findings, including sensory disturbance or ataxia, and did not report vomiting and vertigo. She had no family history suggestive of connective tissue disorders such as Marfan syndrome. Blood tests showed no specific abnormal findings in blood count and the coagulation systems, nor were there any indications of vasculitis. We did not administer anti-platelet therapy and followed up with pain control. Considering the effects on the blood vessels, we did not resume galcanezumab and initiated amitriptyline to control the attack of migraine. For the same reason, we switched from triptan to lasmiditan for the acute treatment of migraine. Her neck pain and headache were relieved, and MRA conducted two months later suggested a complete resolution of the dissection.

### FAERS database analysis

FAERS was downloaded from the FDA website on June 19, 2023 (https://fis.fda.gov/extensions/FPD-QDE-FAERS/FPD-QDE-FAERS.html). We reviewed the publicly available FAERS database from the first quarter of 2012 through to the fourth quarter of 2023, removing duplicate reports (with the same CASE ID number) [6], to search for reports of CeAD as AEs with CGRP mAb (galcanezumab, fremanezumab, erenumab, and eptinezumab). Adverse events in the FAERS are registered based on the Medical Dictionary for Regulatory Activities (MedDRA) developed by the International Conference on Harmonization of Technical Requirements for Registration of Pharmaceuticals for Human Use. To detect adverse event names, MedDRA version 26.0 was used. We extracted and analyzed the Preferred Terms (PTs) related to CeAD from the High-level Terms (HLTs), “central nervous system aneurysms, and dissections” (Supplementary Table 1 [see Additional file 1]). However, the PTs did not distinguish between intra- and extra-cranial artery dissection, therefore, we analyzed AEs that included both of cerebral artery dissection and CeAD. To evaluate whether the effect was attributable to the CGRP mAbs themselves rather than migraine, we also searched for sumatriptan, a widely prescribed drug for migraine, as a comparator. A total of 13,290,393 AE reports were submitted to the FAERS, including 20,946 reports on galcanezumab, 69,906 reports on CGRP mAbs, and 33,462 reports on sumatriptan. Six cases of cerebral artery dissection and CeAD AEs were reported with galcanezumab and 10 cases with CGRP mAbs (Table 1). According to the disproportionality analysis [7], the reporting odds ratios (RORs) for galcanezumab and CGRP mAbs in the FAERS for cerebral artery dissection and CeAD were elevated to 14.0 (95% confidence interval [CI]: 6.22–31.4) and 7.06 (95% CI: 3.75–13.3), respectively. On the other hand, the ROR for sumatriptan was not significantly elevated (2.87; 95% CI: 0.71–11.5).

**Table 1:** The number of patients reported with cerebral artery dissection and CeAD as adverse effects, and ROR (95% CI) in the FAERS database (total *n* = 13,290,393)

Integrated PTs (number of total reports)	Galcanezumab (*n* = 20,946)		CGRPs (*n* = 69,906)		Sumatriptan (*n* = 33,462)	
	*n*	ROR (95% CI)	*n*	ROR (95% CI)	*n*	ROR (95% CI)
PTs of cerebral artery dissection and CeAD (278)	6	14.0 (6.22–31.4)	10	7.06 (3.75–13.3)	2	2.87 (0.71–11.5)

Data are reported as frequencies along with the ROR and 95% CI. The total sample size was 13,290,393. CeAD, cervical artery dissection; ROR, reporting odds ratio; CI, confidence interval; FAERS, food and drug administration adverse event reporting system; PT, preferred term; CGRP, calcitonin gene-related peptide

## Discussion and conclusions

Migraine and cervical artery dissection (CeAD) have been suggested to be associated. Many studies have reported an association between migraine and ischemic stroke (IS); patients with any type of migraine were reported to have a 2.04 (95% CI: 1.72–2.43) times higher risk of IS, with a particularly elevated risk of 3.65 (95%CI: 2.21–6.04) in women under 45 years old [8, 9]. The association between migraine and CeAD has been suspected to be one of the factors increasing IS in patients with migraine [9]. A systematic review found that patients with migraine had a 1.74 times higher risk of developing CeAD [2]. Metso et al. reported that patients with IS and CeAD had a higher frequency of migraine without aura compared to patients with IS from other causes [10]. Recent genetic analyses have reported an association between migraines and CeAD; a genetic correlation study of pairwise traits identified *ADAMTSL4/ECM1*, *PLCE1*, and *MRVI1* as new candidate genes implicated in the susceptibility to both migraine and CeAD [3]. Migraine can, in rare instances, lead to mild ischemic cerebrovascular deficits with a relatively benign prognosis [11]. Although our case also had a benign prognosis, the absence of ischemic infarction suggests that this particular scenario may not apply to our case.

CGRP is an essential multifunctional neuropeptide discovered in 1982 as one of the first examples of alternative RNA processing [12]. Since then, a series of researches have revealed the role of CGRP in the cranial sensory nerves associated with migraines, and multiple CGRP transmission components are targeted as migraine therapies [4]. CGRP is one of the most potent vasodilators in humans, which increases cerebral, cardiac, and renal blood flow [13, 14]. CGRP is released endogenously in response to ischemia and has been suggested to play a role in preconditioning and protection against reperfusion injury of the brain and various organs [15]. CGRP mAb inhibit these effects, thereby potentially increasing the risk of cardiovascular events. Mulder et al. reported that administering gepant, a CGRP receptor antagonist, to mice and inducing artificial vascular occlusion resulted in a significantly higher incidence and extent of cerebral infarction compared with vehicle [16]. Currently, no clinical evidence suggests an increased risk of cerebrovascular events associated with CGRP mAb. However, the European Headache Federation guidelines recommend cautious use of CGRP mAb in patients with high cerebrovascular risk [17]. Although there is no specific hypothesis regarding the association between CGRP mAb and CeAD, the mechanism of the CGRP effect suggests that it may affect CeAD as well as cerebral infarction. However, there is no evidence of a strong correlation between CeAD and CGRP.

We investigated the number of cerebral artery dissection and CeAD events reported as AEs of CGRP mAb, using the FAERS database, which was utilized in the previous study to report the adverse event profile of CGRP mAb, including cases of coronary artery dissection (*n* = 5) [18]. The RORs for galcanezumab and CGRP mAbs compared with all the other drugs in FAERS for cerebral artery dissection and CeAD were significantly elevated. However, cerebral artery dissection and CeAD are more likely to occur in patients with migraine; [2] therefore, these results may be indicative of the characteristics of the population of migraine patients. In contrast, sumatriptan did not show a significantly elevated ROR, suggesting that CGRP mAb themselves may increase the risk of developing cerebral arterial dissection and CeAD. Further high-quality evidence is necessary to determine whether CGRP mAb is associated with cerebral arterial dissection and CeAD.

Several limitations should be noted. First, our case developed vertebral artery dissection long after the initiation of galcanezumab, so the association between them is unclear. Second, the FAERS database is voluntary reporting system and includes various biases, such as reporting heterogeneity, population background, and disease prevalence. Therefore, results from the FAERS database analysis do not necessarily represent a causal relationship. However, our report is the first to examine the relationship between CGRP mAb and CeAD. We propose conducting a large-scale safety investigation of CGRP mAb in relation to vascular events, as well as an in vivo study to evaluate their effects on the vasculature and explore potential connections.

In summary, we describe a case of vertebral artery dissection in a patient with migraine who received CGRP mAb treatment for more than one year. It is unclear whether vertebral artery dissection and the use of CGRP mAb are causally related. To the best of our knowledge, however, our case report is the first focusing on CeAD and CGRP mAb. Considering the characteristics of CGRP and the result of FAERS database analysis, the potential for CGRP mAb to be related to CeAD cannot be ruled out. Further case series and studies are required to validate the association between CGRP mAb and CeAD.

## Electronic supplementary material

Below is the link to the electronic supplementary material.

Supplementary Table 1

## Data Availability

No datasets were generated or analysed during the current study.

## Abbreviations

CeAD: Cervical artery dissection
CGRP: Calcitonin gene-related peptide
CGRP mAb: Anti-calcitonin gene-related peptide monoclonal antibodies
FDA: Food and drug administration
FAERS: Food and drug administration adverse event reporting system
AEs: Adverse effects
MedDRA: Medical dictionary for regulatory activities
PTs: Preferred terms
HLTs: High-level terms
RORs: Reporting odds ratios

## References

[1] Steiner TJ, Stovner LJ. Global epidemiology of migraine and its implications for public health and health policy. Nat Rev Neurol. 2023;19(2):109–17. 36693999 10.1038/s41582-022-00763-1

[2] Sun Z, Kleine-Borgmann J, Suh J, McDermott GC, Vishnevetsky A, Rist PM. Migraine and the risk of cervical artery dissection: a systematic review and meta-analysis. Eur Stroke J. 2023;8(4):904–14. 37555306 10.1177/23969873231191860 PMC10683742

[3] Daghals I, Sargurupremraj M, Danning R, Gormley P, Malik R, Amouyel P, et al. Migraine, Stroke, and cervical arterial dissection: Shared Genetics for a Triad of Brain disorders with vascular involvement. Neurol Genet. 2022;8(1):e653. 35128049 10.1212/NXG.0000000000000653 PMC8808356

[4] Russell FA, King R, Smillie SJ, Kodji X, Brain SD. Calcitonin gene-related peptide: physiology and pathophysiology. Physiol Rev. 2014;94(4):1099–142. 25287861 10.1152/physrev.00034.2013 PMC4187032

[5] Ray JC, Kapoor M, Stark RJ, Wang SJ, Bendtsen L, Matharu M, et al. Calcitonin gene related peptide in migraine: current therapeutics, future implications and potential off-target effects. J Neurol Neurosurg Psychiatry. 2021;92(12):1325–34. 33495299 10.1136/jnnp-2020-324674

[6] Wu B, Hu Q, Tian F, Wu F, Li Y, Xu T. A pharmacovigilance study of association between proton pump inhibitor and dementia event based on FDA adverse event reporting system data. Sci Rep. 2021;11(1):10709. 34021217 10.1038/s41598-021-90108-7 PMC8139970

[7] Rothman KJ, Lanes S, Sacks ST. The reporting odds ratio and its advantages over the proportional reporting ratio. Pharmacoepidemiol Drug Saf. 2004;13(8):519–23. 15317031 10.1002/pds.1001

[8] Spector JT, Kahn SR, Jones MR, Jayakumar M, Dalal D, Nazarian S. Migraine headache and ischemic stroke risk: an updated meta-analysis. Am J Med. 2010;123(7):612–24. 20493462 10.1016/j.amjmed.2009.12.021 PMC2900472

[9] Schurks M, Rist PM, Bigal ME, Buring JE, Lipton RB, Kurth T. Migraine and cardiovascular disease: systematic review and meta-analysis. BMJ. 2009;339:b3914. 19861375 10.1136/bmj.b3914 PMC2768778

[10] Metso TM, Tatlisumak T, Debette S, Dallongeville J, Engelter ST, Lyrer PA, et al. Migraine in cervical artery dissection and ischemic stroke patients. Neurology. 2012;78(16):1221–8. 22491867 10.1212/WNL.0b013e318251595f

[11] Arboix A, Massons J, García-Eroles L, Oliveres M, Balcells M, Targa C. Migrainous cerebral infarction in the Sagrat Cor Hospital of Barcelona stroke registry. Cephalalgia. 2003;23(5):389–94. 12780770 10.1046/j.1468-2982.2003.00534.x

[12] Amara SG, Jonas V, Rosenfeld MG, Ong ES, Evans RM. Alternative RNA processing in calcitonin gene expression generates mRNAs encoding different polypeptide products. Nature. 1982;298(5871):240–4. 6283379 10.1038/298240a0

[13] Brain SD, Williams TJ, Tippins JR, Morris HR, MacIntyre I. Calcitonin gene-related peptide is a potent vasodilator. Nat 1985;313(5997):54–6. 10.1038/313054a0 3917554

[14] Brain SD, Grant AD. Vascular actions of calcitonin gene-related peptide and adrenomedullin. Physiol Rev. 2004;84(3):903–34. 15269340 10.1152/physrev.00037.2003

[15] Kee Z, Kodji X, Brain SD. The role of calcitonin gene related peptide (CGRP) in Neurogenic Vasodilation and its cardioprotective effects. Front Physiol. 2018;9:1249. 30283343 10.3389/fphys.2018.01249 PMC6156372

[16] Mulder IA, Li M, de Vries T, Qin T, Yanagisawa T, Sugimoto K, et al. Anti-migraine calcitonin gene-related peptide receptor antagonists worsen cerebral ischemic outcome in mice. Ann Neurol. 2020;88(4):771–84. 32583883 10.1002/ana.25831 PMC7540520

[17] Sacco S, Amin FM, Ashina M, Bendtsen L, Deligianni CI, Gil-Gouveia R, et al. European Headache Federation guideline on the use of monoclonal antibodies targeting the calcitonin gene related peptide pathway for migraine prevention – 2022 update. J Headache Pain. 2022;23(1):67. 35690723 10.1186/s10194-022-01431-x PMC9188162

[18] Wang Q, Liu J, Sun H, Dong Y, Tan W, Tang Z, et al. Adverse event profile of CGRP monoclonal antibodies: findings from the FDA adverse event reporting database. Expert Opin Drug Saf. 2024;23(1):107–17. 37720989 10.1080/14740338.2023.2250720

