# Correction: Vertebral artery dissection in a patient with migraine treated with calcitonin gene-related peptide monoclonal antibody: a case report and FAERS database analysis

**DOI:** 10.1186/s12883-025-04052-4

**Published:** 2025-01-28

**Authors:** Daiki Tokuyasu, Shungo Imai, Shih-Pin Chen, Keiko Ihara, Narumi Watanabe, Yoshikane Izawa, Jin Nakahara, Satoko Hori, Tsubasa Takizawa

**Affiliations:** 1 Department of Neurology, Keio University School of Medicine, 35 Shinanomachi, Shinjuku-ku, Tokyo, 160-8582 Japan; 2 Division of Drug Informatics, Keio University Faculty of Pharmacy, Tokyo, Japan; 3 Division of Translational Research, Department of Medical Research, Taipei Veterans General Hospital, Taipei, Taiwan; 4 Institute of Clinical Medicine, College of Medicine, National Yang Ming Chiao Tung University, Taipei, Taiwan; 5 Brain Research Center, National Yang Ming Chiao Tung University, Taipei, Taiwan; 6 Japanese Red Cross Ashikaga Hospital, Ashikaga, Japan

**Correction: BMC Neurol 25, 1 (2025)**

10.1186/s12883-024-04009-z

Following publication of the original article [1], the authors identified an error in the author name of Shih-Pin Chen.

The incorrect author name is: Shin-Pin Chen

The correct author name is: Shih-Pin Chen

The author group has been updated above and the original article [1] has been corrected.
