# Supplementary material for: Vertebral artery dissection in a patient with migraine treated with calcitonin gene-related peptide monoclonal antibody: a case report and FAERS database analysis
Source: BMC Neurol. 2025 Jan 2;25:1. doi: 10.1186/s12883-024-04009-z (PMC11694463; doi:10.1186/s12883-024-04009-z)
Supplement: Supplementary file 1 — Supplementary Table 1 [file 12883_2024_4009_MOESM1_ESM.docx]

**Supplementary Table1. List of Preferred Terms used in our analysis**

Out of the Preferred Terms (PTs) listed in “High-Level Terms 10074471/Central nervous system aneurysms and dissections”, PTs of “Carotid artery dissection”, “Intracranial artery dissection”, “Precerebral artery dissection” and “Vertebrobasilar artery dissection” (shown in bold) are related to “cervical artery dissection”. In this study, we combined and analyzed these PTs as “cerebral artery dissection and cervical artery dissection (CeAD)”.

| HLT | 10074471/Central nervous system aneurysms and dissections | |
| --- | --- | --- |
|  | PT | 10007686/Carotid artery aneurysm |
|  | PT | 10008076/Cerebral aneurysm ruptured syphilitic |
|  | PT | 10022758/Intracranial aneurysm |
|  | PT | 10048661/Wyburn Mason's syndrome |
|  | PT | **10050403/Carotid artery dissection** |
|  | PT | 10054749/Charcot-Bouchard microaneurysms |
|  | PT | **10073565/Intracranial artery dissection** |
|  | PT | 10077498/Vertebral artery aneurysm |
|  | PT | 10077607/Basilar artery aneurysm |
|  | PT | 10086097/Precerebral artery aneurysm |
|  | PT | **10086098/Precerebral artery dissection** |
|  | PT | **10087672/Vertebrobasilar artery dissection** |
